# Effectiveness of a Health Education Program in Hypertensive Patients with Dyslipidemia and/or Microalbuminuria: A Quasi-Experimental Study in Vinh Long Province, Vietnam

**DOI:** 10.3390/healthcare11152208

**Published:** 2023-08-04

**Authors:** Minh Huu Le, Trung Kien Nguyen, Thi Tam Pham, Trung Tin Pham, Van De Tran

**Affiliations:** 1 Department of Epidemiology, Can Tho University of Medicine and Pharmacy, Can Tho 900000, Vietnam; lmhuu@ctump.edu.vn; 2 Department of Physiology, Faculty of Medicine, Can Tho University of Medicine and Pharmacy, Can Tho 900000, Vietnam; ntkien@ctump.edu.vn; 3 Department of Nutrition and Food Safety, Can Tho University of Medicine and Pharmacy, Can Tho 900000, Vietnam; pttam@ctump.edu.vn; 4 Department of Health Organization and Management, Can Tho University of Medicine and Pharmacy, Can Tho 900000, Vietnam

**Keywords:** hypertension, non-professional educator, BMI, medication adherence, dyslipidemia, microalbuminuria

## Abstract

Introduction: Hypertension, a major health concern, is associated with significant mortality and disease burden worldwide, including Vietnam. Comprehensive interventions targeting medication, lifestyle modifications, dyslipidemia (DLP), and microalbuminuria (MAU) are vital for effective hypertension management and reducing the risk of cardiovascular disease complications (CDV). While medication interventions have proven efficacy, the evidence regarding the effectiveness of community-based health education interventions in managing DLP and MAU is limited. Therefore, this study aims to evaluate the effectiveness of community health education interventions in reducing hypertension risk factors and achieving hypertension management objectives, as well as managing DLP and MAU among hypertension patients. Methods: A quasi-experimental study was conducted on 330 hypertensive patients with dyslipidemia (DLP) and/or microalbuminuria (MAU) who were divided into a control group (n = 164) and an intervention group (n = 166). The control group received standard national hypertension management, while the intervention group received additional intensive health education provided by trained volunteers. The effectiveness of the intervention was assessed by comparing outcomes such as lifestyle factors, BMI control, treatment adherence, hypertension control, and DLP and MAU status between the two groups before and after a two-year intervention period. Results: The health education intervention resulted in significant reductions in dietary risk factors, specifically in fruit and vegetable consumption (*p* < 0.001). There was a lower prevalence of high salt intake in the intervention group compared to the control group (*p* = 0.002), while no significant differences were observed in other dietary factors. Smoking habits and low physical activity significantly decreased in the intervention group, with a notable disparity in physical activity proportions (*p* < 0.001). Both groups showed significant improvements in achieving hypertension management targets, with the intervention group demonstrating superior outcomes. The intervention was effective in reducing the prevalence of risk factors, particularly treatment non-adherence, blood pressure control, and low physical activity. Additionally, the intervention group had a higher likelihood of achieving DLP and MAU control compared to the control group. Conclusions: This study underscored the additional positive impact of incorporating health education by non-professional educators in achieving favorable outcomes, including better control of BMI, blood pressure, medication adherence, and management of dyslipidemia (DLP) and microalbuminuria (MAU). Further research is warranted to fully explore the potential of health education in primary healthcare settings and maximize its effectiveness.

## 1. Introduction

Hypertension carries significant importance within the realm of global health, given its profound impacts and repercussions. It is estimated to contribute to 7.5 million deaths worldwide, accounting for approximately 12.8% of total global mortality. Furthermore, hypertension adds to the disease burden, accounting for 57 million DALYs (3.7% of global DALYs) [1]. Among adults in low- and middle-income countries (LMICs), the prevalence of hypertension is 31.1%, with a treatment rate of 44.5%, and only 17.9% achieving optimal blood pressure control [2]. Notably, in Vietnam, the prevalence of hypertension among adults aged 18 and older was recorded at 47.3% [3]. Pharmacological and non-pharmacological interventions are recommended to address hypertension and associated cardiovascular disease (CVD) complications [4,5].

Several important aspects that deserve attention in hypertension management are the prevention and management of complications associated with cardiovascular disease (CVD). Firstly, an elevated risk for CVD complications is posed by the presence of dyslipidemia (DLP) and/or microalbuminuria (MAU) in combination with hypertension [6,7,8]. Addressing these conditions with medication intervention is well-documented. In the case of dyslipidemia, several reviews on medication approaches highlighted the efficacy of pharmacological interventions [9,10]. Likewise, successful MAU management with medication was proved in some trials [11,12]. Considering DLP and MAU management as part of comprehensive management strategies for hypertension, healthcare professionals can mitigate the risk of CVD complications and improve patient outcomes.

In addition to medication, lifestyle modifications are recommended for controlling blood pressure and managing DLP and MAU [13,14]. Nevertheless, the evidence regarding their efficacy in addressing DLP and MAU in a community setting is scarce. Few studies have been able to provide substantial evidence regarding the effectiveness of interventions in achieving their goals [15,16,17]. There is also limited evidence regarding the effectiveness of community health education in promoting lifestyle modifications for DLP and MAU management purposes. This research aimed to assess the effectiveness of community health education interventions in reducing hypertension risk factors and managing hypertension objectives, DLP, and MAU among patients. The results of this study might provide insight for policy makers in terms of implementing mass health education programs.

## 2. Methods

### 2.1. Study Design

A quasi-experimental study was conducted on a total of 330 hypertensive patients with DLP and/or with MAU. These patients were randomly selected from the hypertensive patient management list of commune clinics in two districts of Vinh Long province, Vietnam. They were then allocated into two groups: a control group consisting of 164 patients and an intervention group consisting of 166 patients. The control group (164 patients) received the standard national hypertension management program, while the intervention group (166 patients) received the same program supplemented with an intensive health education program delivered by trained volunteers during the two-year intervention period. The effectiveness of the intervention was assessed by comparing the rates of outcomes including lifestyle factors, BMI control, treatment adherence, hypertension control, and DLP and MAU status between the two groups before and after the 2-year intervention, from December 2016 to December 2018.

### 2.2. Setting

In Vietnam’s national strategy for noncommunicable disease prevention and control until 2025, holistic measures address cardiovascular diseases and hypertension. These include activities focused on leadership, policy implementation, and coordination among relevant departments and agencies. Efforts strengthen the capacity of healthcare facilities and personnel in preventing, detecting, managing, treating, and caring for cardiovascular diseases and hypertension. Specialized programs and plans address tobacco and alcohol harms and promote physical activity at the commune level, the lowest administrative divisions in Vietnam. Salt reduction campaigns are also implemented to prevent hypertension and stroke. For instance, the program targets individuals aged forty and above with a considerable risk of hypertension. Population screening identifies high blood pressure, ensures medication coverage through health insurance at the commune level, and provides hypertension prevention education led by lay health workers [18]. Those measures are currently implemented in both districts of this study.

### 2.3. Sampling

The sampling process in this study involved multiple stages, including randomization and systematic random sampling techniques, and was conducted in Vinh Long province. Firstly, two districts, Binh Minh and Mang Thit, were randomly selected from a population of eight districts in Vinh Long province. Within each of the selected districts, four wards were randomly chosen. The sampling frame for this study consisted of the hypertensive patient management list from the respective wards, comprising a total of 778 patients. From this sampling frame, all patients with DLP and/or MAU were identified based on their health records. These selected patients formed the eligible pool for inclusion in the study. Then, utilizing a systematic random sampling approach resulted in 170 patients for each arm of the study in the sampling section. Within each of the four selected wards, around 40 patients with DLP and/or MAU were systematically chosen from the sampling frame. Moreover, patients from the Binh Minh district were assigned to the intervention group, while patients from the Mang Thit district were assigned to the control group. This assignment aimed to examine the effectiveness of the intervention on the outcome variables, comparing it to the control condition.

Participants met the study’s inclusion criteria for hypertension diagnosis based on the European Society of Hypertension guidelines: systolic BP ≥ 140 mmHg and/or diastolic BP ≥ 90 mmHg, or current use of antihypertensive medication, with evidence of current DLP and/or MAU [19]. Participants needed to have at least six months of residency in Vinh Long province and provide informed consent to be included in the study.

Exclusion criteria encompassed individuals who relocated during the study, those with communication impairments from psychiatric disorders, severe mobility limitations, end-stage renal disease, pregnant women with hypertension, and individuals with a history of myocardial infarction or stroke. The loss to follow-up rates were 3.5% (6 out of 170) in the intervention group and 2.35% (4 out of 170) in the control group. These rates, below the accepted threshold of 5%, suggest minimal potential for bias due to loss to follow-up, although it cannot be excluded [20].

### 2.4. Measurements 

Baseline data, including age, gender, educational level, marital status, health insurance status, and time of hypertension diagnosis, were collected by trained interviewers. Additionally, in this research, various measurements were taken to assess the effectiveness of the intervention. They were primary and secondary outcomes measured both before and after the intervention. Secondary outcomes were lifestyle risk factors and treatment objectives at baseline time and after the two-year period. Firstly, the risky lifestyle habits included low consumption of vegetables (under 400 g/day), high salt intake (greater than 3 days/week), high consumption of fried or processed foods (greater than 3 days/week), high alcohol consumption with over 5 drinks/week for men and 2 drinks/week for women (yes/no), low physical activity level with under 150 min of moderate physical activity/week (yes/no), and tobacco smoking habits (yes/no). These outcomes were measured by self-reporting questions. Secondly, weight control (BMI < 23), adherence to treatment protocols (Morisky—8 questions adherence score <2), and blood pressure control (systolic BP < 140 mmHg and diastolic BP < 90 mmHg) were other important secondary outcomes, which served as objectives of hypertension management. The primary outcome in this intervention research pertained to lipid profile control (Yes/No) and MAU status (Yes/No). Achieving DLP (Yes) was determined by meeting specific criteria for all of the four lipid components: total cholesterol < 5.2 mmol/L, triglyceride < 1.7 mmol/L, LDL-C < 3.4 mmol/L, and HDL-C ≥ 1 mmol/L. If any of these components did not meet the desired targets, the participant was considered as not achieving DLP (No). MAU control was assessed based on the albumin-to-creatinine ratio (ACR) being below 30. On the other hand, achieving MAU control (Yes) was determined by attaining an albumin-to-creatinine ratio (ACR) below 30, while patients with ACR > 30 were considered not achieving the target (No).

The test procedure of each patient was performed by commune clinic nurses collaborating with the laboratory department of Can Tho University of Medicine and Pharmacy. To calculate the cost-effectiveness of the intervention, the Incremental Cost-Effectiveness Ratio (ICER) was used. Based on the formula for ICER, the cost difference between the two groups was the additional cost incurred for the health education activities. The direct costs include payments for the intervention team, health education activities, training activities, and management payments to the healthcare station. The indirect costs are assumed to be the same between the two groups.

### 2.5. Data Analysis

Epidata software was used for data entry, and SPSS software was used for data analysis on 164 patients in the intervention group and 166 patients in the control group. Frequency distributions were utilized to describe the sociodemographic characteristics of both the intervention and control groups. At baseline, homogeneity between the two groups across demographic variables, lifestyle factors related to hypertension, and hypertension history was assessed using chi-square tests. Similarly, the chi-square test was employed to compare the differences in secondary and primary outcomes between the control and intervention groups prior to the intervention. A significance level of 0.05 was used for all tests to establish statistical significance. The effectiveness of the intervention was measured both within groups and between groups. Within each group, the McNemar test was employed to assess the differences in all outcomes before and after the intervention. The chi-square test was utilized to assess the differences between the intervention group and the control group after the intervention. The effectiveness of the intervention was evaluated by assessing changes within each group before and after the intervention, as well as differences observed between the groups after the intervention. Moreover, to estimate the effectiveness of dependent intervention in terms of mitigating risk factors, absolute risk reduction (ARR) was also calculated. The number needed to treat (NNT) was calculated to quantify effectiveness by determining the number of patients needed to receive the intervention for risk factor reduction in one patient. The difference between groups was measured for each specific outcome in terms of risk behaviors and treatment goals, such as achieving blood pressure, DP, and MAU targets. Since multiple outcomes were measured, the total cost was assumed to be evenly distributed among the different outcomes, and the ICER was calculated accordingly.

### 2.6. Intervention

Health education for managing DLP and/or MAU in hypertension followed key principles: community-based implementation, addressing healthcare needs, minimizing monetary impact, ensuring simplicity and consistency, aligning with local conditions, and providing clear information. 

Initially, based on baseline lipid profile tests, patients in both groups were informed about their condition and advised on treatment options at a medical station or a preferred location of their choice. Each patient in intervention groups was introduced to the health education activities provided by a volunteer team for the next 2 years.

In each selected ward, the intervention team consisted of volunteer students in public health and preventive medicine, along with a local primary care physician. The trained students conducted health education sessions for patients and their families in the intervention group, supported by a primary physician from each commune health station. The team received counseling support for health education activities from supervisors and faculty members at Can Tho University of Medicine and Pharmacy.

Two voluntary students were assigned to each ward, with approximately forty patients. They conducted group health education sessions at community centers or suitable venues in January, March, June, and December of the first year. During the sessions, volunteers provided additional guidance to hypertensive patients regarding non-pharmacological treatment measures, including smoking cessation, alcohol reduction, balanced diet, weight management, treatment adherence, and regular blood pressure monitoring.

Additionally, the team also conducted monthly household visits to support and monitor patients and their family members. Each patient was individually assigned specific targets tailored to their current lifestyle and hypertension condition. Health education objectives were tailored to each patient’s needs, covering medication adherence, physical activity, diet, weight management, and blood pressure control. Family members were provided with educational materials to support and remind patients to follow recommendations. During subsequent household visits, the team concentrated on addressing any unmet targets by collaboratively fostering feasible health behaviors for individuals with hypertension. This approach aimed to help patients gradually achieve their desired outcomes.

The health education content aimed to empower hypertensives to actively manage their blood pressure and make positive lifestyle changes by providing concise and easily understandable knowledge and instructions. The content emphasized risks, smoking and alcohol impact, dietary guidelines, and physical activity’s significance for hypertension control. Local terminology and examples were used to enhance comprehension and implementation.

## 3. Results

### 3.1. Groups Comparison

To assess group homogeneity at baseline, a chi-square test examined demographic characteristics, medical records, lifestyle factors, hypertension management, and DLP and MAU status.

The study found a higher proportion of females than males in both groups (59.1% in the intervention group, 62.7% in the control group). The most common age group was 60–69 years, comprising 47% in the intervention group and 50% in the control group. Notably, primary education was the highest educational level, comprising over 40% in both groups. The results of the chi-square test indicated a similarity in sociodemographic characteristics, including gender, age, ethnicity, and education level, between the intervention and control groups (*p* > 0.4) (Table 1). Likewise, the study found no significant differences between both groups in terms of duration of hypertension and comorbidities with all *p*-values greater than 0.3 (Table 2). In both groups, most patients, accounting for around 60%, had a diagnosis of hypertension for less than 5 years. The prevalence of concomitant conditions, such as cardiovascular disease or diabetes mellitus, was below 20% in both groups. The study also found no significant difference between the two groups in the proportion of having DLP and/or MAU with *p* = 0.999.

Table 3 indicates that three common lifestyle risk factors were prevalent in both study groups: low fruit and vegetable intake, high salt consumption, and low physical activity, with over 60% in all groups. The study found that most participants had uncontrolled blood pressure, with rates of 67.7% and 70.5% in the intervention and control groups, respectively. No significant differences were found between the two groups in terms of lifestyle-related risk factors, BMI control, treatment adherence, and blood pressure control. 

### 3.2. Intervention Results

Table 4 illustrates the means and standard deviations of measurable outcomes before and after intervention. In terms of propotion, after the intervention, significant reductions in all dietary risk factors were observed in the intervention group (*p* < 0.001). Dissimilarly, a significant decrease was only noted in the consumption of fruits and vegetables (*p* < 0.001), while the others showed insignificant changes. The prevalence of high salt intake was significantly lower in the intervention group compared to the control group (*p* = 0.002, ARR = 16.9%, NNT = 6). However, there were no significant differences in the other dietary factors between the two groups (Table 5).

The intervention group showed significant reductions in smoking habits (*p* = 0.027) and low physical activity levels (*p* < 0.001) compared to baseline. However, no significant changes were observed in the control group for these factors. Furthermore, Table 4 shows that the disparity of low physical activity proportion was significant (*p* < 0.001, ARR = 21.9%, NNT = 5). However, there was no significant change in alcohol consumption and smoking habits between both groups after the intervention (*p* > 0.05).

Both groups showed significant improvements in achieving hypertension management targets, including BMI control (*p* < 0.001, *p* = 0.02), treatment adherence (*p* < 0.001, *p* = 0.01), and blood pressure control (*p* < 0.001, *p* = 0.02) at the endpoint (Table 6). Noticeably, the intervention group exhibited superior outcomes compared to the control group (BMI controlled: *p* = 0.024; treatment adherence: *p* < 0.001; BP control: *p* < 0.001). The study results demonstrated that independent interventions could reduce the prevalence of certain risk factors. The highest absolute risk reductions (ARRs) were observed for treatment non-adherence (ARR = 31.3%, NNT = 3), blood pressure control (ARR = 27.9%, NNT = 3), and low physical activity (ARR = 21.9%, NNT = 5). The ARR values in other risk factor groups ranged from 6.5% to 16.9%. 

Regarding the DLP control goal, Table 7 shows that the intervention group had a higher chance of achieving the goals of managing these conditions compared to the control group (DLP: RR = 1.71, 95% CI: 1.12–2.7, *p* = 0.012). The independent health education intervention reduced the proportion of patients with DLP by 11.6% over 2 years, with an NNT of approximately nine patients. Likewise, the intervention group also demonstrated a greater likelihood of effectively managing MAU when compared to the control group (RR = 1.52, CI 95%, *p* = 0.007). ARR for microalbuminuria was 17.9% with an NNT of around six patients, respectively.

Table 8 shows that implementing health education activities over a span of 2 years can help reduce certain risky behaviors associated with hypertension and urinary protein levels. Among the risky behaviors, the cost to increase patient adherence to treatment and decrease low physical activity levels is the lowest, at USD 13 and USD 18, respectively. Notably, the cost to achieve blood pressure targets per patient is USD 15, which is lower than the cost to achieve targets for dyslipidemia and urinary protein levels, which is USD 34. Additionally, the Incremental Cost-Effectiveness Ratio (ICER) is highest for alcohol consumption and smoking habits, at USD 152 per behavior.

## 4. Discussion

The study utilized a quasi-experimental design with equivalent control and intervention groups in terms of sociodemographic characteristics, disease characteristics, and risk behaviors. This study’s strength lies in the similarity of the two groups, which was essential for evaluating the true effectiveness of the intervention on outcomes. Furthermore, this study design allowed for the use of ARR and NNT indices to assess intervention effectiveness, providing valuable evidence for policy makers to adjust health intervention strategies [21,22]. Future studies should evaluate the cost-effectiveness of additional health education to assess its benefits.

This study differs from previous studies in terms of the implementation of health education regarding the venue and health educator. Some previous studies utilized healthcare professionals such as public health experts, nurses, and physicians to conduct health education activities at healthcare facilities [23,24,25]. It can be observed that the effectiveness of health education may vary, partly depending on the capacity of the health educators. Previous studies assessed healthcare staff self-efficacy, but no comparisons were made between healthcare professionals and non-professionals [26]. In this study, health education activities were conducted by students. Utilizing trained non-professional healthcare personnel, such as volunteer students, in the intervention proved effective in achieving desired outcomes, highlighting the effectiveness of health education. Future studies should assess the capacity and availability of health educators, especially among non-professionals, to determine the sustainability of health education interventions.

In this study, the results showed that the additional health education intervention was statistically effective in reducing the salt intake rate. However, no significant differences were found in the rates of consuming fewer vegetables and consuming high-fat foods between the two groups after the intervention. Regarding salt intake, previous studies also demonstrated the effectiveness of health education interventions in reducing salt consumption [27,28]. Interestingly, the two risk behaviors of consuming fewer vegetables and consuming high-fat foods yielded contrasting results. The control group showed a significant decrease in vegetable consumption but no significant change in high-fat food intake. These results suggest that existing healthcare programs may be effective in promoting vegetable consumption but may not be enough to address the habit of consuming fried, fatty foods and high salt intake. Our results are different from a system review study which showed success in salt intake with various interventions and measurements [29]. The lack of effectiveness in reducing salt intake and the consumption of fried, fatty foods can be attributed to several factors. Firstly, variations in measurement methods across studies make it challenging to compare and evaluate the impact of interventions consistently [30,31]. Secondly, cultural cuisine and family eating habits, including the incorporation of fried vegetables and the use of fish sauce in Vietnamese meals, have a significant influence on dietary practices. For that reason, further studies should conduct experiments to understand the causes and propose solutions to minimize these two risk factors in hypertensive patients.

The intervention did not significantly impact the disparity in alcohol and tobacco use between the two groups after 2 years. Importantly, the rate of harmful alcohol consumption remained unchanged in both groups after the intervention. These results are not consistent with the study of Felicia W Chi, which achieved the objectives of reducing alcohol consumption [32,33]. These findings highlight the challenges of addressing addictive substances through health education, as it requires specialized skills and a supportive environment that may exceed the capabilities of the intervention groups [33,34]. Therefore, subsequent research on interventions aiming to reduce alcohol consumption and smoking in low-resource settings requires attention. By addressing challenges in low-resource settings, interventions can effectively promote behavior change and reduce alcohol- and smoking-related harms for hypertensive individuals.

When evaluating the effectiveness of the intervention in achieving outcomes such as BMI control, treatment adherence, and blood pressure control, the intervention group showed statistically significant differences in all three aspects. These results align with previous studies that have demonstrated improved effectiveness in reducing blood pressure or achieving blood pressure control [35,36,37]. Overall, the intervention in this study led to significant improvements in all outcome measures, successfully achieving the desired outcomes in hypertension treatment. These findings align with previous research highlighting the relationship between health literacy, BMI control, and hypertension control [38,39]. This serves as evidence for implementing additional community health education activities with non-professional resources which might enhance the outcomes of hypertensives with affordable resources. 

Furthermore, the effectiveness of the intervention is further supported by the increased rate of DLP and MAU control in the intervention group. Previous studies have only shown the effectiveness of intervention in dyslipidemia when using medication or lifestyle modifications, but the rate remains low [17,40]. Regarding microalbuminuria management, our results are consistent with previous studies that highlight lifestyle factors as risk factors for microalbuminuria [41,42,43,44]. Our findings partly suggest a correlation between lifestyle improvement and the improvement in the microalbuminuria rate. The effectiveness of our study suggests the feasibility of community-based interventions after a 2-year period. Further studies should investigate the enablers and barriers to non-pharmacological interventions and consider the time required to achieve effectiveness.

The estimated ICER results suggest that health education has varying effectiveness for different outcomes. This difference indicates that health education programs should focus on behaviors that are modifiable and have lower costs. Risky behaviors such as smoking and alcohol consumption may require consideration of alternative indirect measures such as changes in pricing policies or taxes.

### Limitations

The first limitation was that the potential bias introduced by our sampling process due to the unavoidable difference in geographic characteristics is acknowledged. To assess this bias, an analysis of the baseline characteristics was conducted between the intervention and control groups, revealing no significant differences. This indicates a minimal risk of bias in our study, although it cannot be excluded. 

Secondly, in this study, the evaluator also functioned as the interventionist, which could have introduced subjectivity in assessing the changes in risk behaviors and other outcomes. However, implementing blind assessment using a separate team of investigators was not practically feasible as the enhanced health education activities would have been easily recognizable. To address potential bias in the evaluation process, the research team enhanced regular training activities and reinforced the honesty of the volunteers. Additionally, a good rapport with participants was established through home visits to foster open and honest responses. The decision not to use different individuals for data collection was made to maintain the established rapport and ensure reliable responses. Finally, in addition to subjective outcomes, we also measured objective outcomes such as automated blood pressure measurements, lipid profile tests, and microalbuminuria in both groups.

Another limitation of the study was that the outcomes were assessed as binary variables for continuous variables such as microalbuminuria and blood pressure control. This may reduce the accuracy in evaluating the true effectiveness of health education activities as seen in quantitative studies. However, this assessment was beneficial in calculating epidemiological indices such as ARR and NNT, which might be meaningful in policy decision-making. Future studies should consider describing both types of outcomes to provide more insights into community interventions. Moreover, further research should consider using strict time-to-event analysis to explore the impact of health education carefully. 

The magnitude of the effectiveness of the health education program delivered by humans in primary care settings was not always consistent. Distinct reasons are subjective assessment and evaluation, inconsistent health education provided by different educators, and variations in the dose of education received by participants. To improve these interventions, solutions can be implemented. These include standardized assessment and evaluation, comprehensive training, quality assurance, a structured curriculum, technology integration, and fostering collaboration among educators. By implementing these solutions, health education interventions can address the limitations and ensure more standardized, effective, and equitable delivery of education to participants.

## 5. Conclusions

In conclusion, the management of hypertension requires a comprehensive approach that encompasses various aspects. This study highlights the effectiveness of additional health education by non-professional personnel in promoting healthier outcomes, including improved control of BMI, blood pressure, medication adherence, and management of dyslipidemia (DLP) and microalbuminuria (MAU). These findings support the integration of health education interventions in hypertension management programs. However, further research is needed to explore the full potential of health education in primary healthcare settings.

## Figures and Tables

**Table 1:** The sociodemographic characteristics of the two groups.

Characteristic		Intervention Group		Control Group		*p* *
		N	%	n	%	
Gender	Woman	97	59.1	104	62.7	0.514
	Man	67	40.9	62	37.3	
Age	<50 years	8	4.9	8	4.8	0.871
	50–59 years	44	26.8	38	22.9	
	60–69 years	77	47.0	83	50.0	
	≥70 years	35	21.3	37	22.3	
Ethnicity	Kinh	159	97.0	163	98.2	0.463
	Other	5	3.0	3	1.8	
Educational level	Under primary education	37	22.6	36	21.7	0.934
	Primary education	68	41.5	74	44.6	
	Secondary school	32	19.5	32	19.3	
	High school and higher	27	16.5	24	14.5	

* Chi-square test.

**Table 2:** The hypertension characteristics in the intervention and control groups.

Characteristic	Intervention Group		Control Group		*p* *
	n	%	N	%	
Duration of hypertension					
<5 years	94	57.3	106	63.9	0.330
5–9 years	45	27.4	32	19.3	
10–14 years	15	9.1	19	11.4	
≥15 years	10	6.1	9	5.4	
Diabetes					
Yes	19	11.6	19	14.1	0.968
No	145	88.4	147	88.9	
Cardiovascular diseases					
Yes	29	17.7	31	18.7	0.815
No	135	82.3	135	81.3	
Dyslipidemia and Microalbuminuria					
Dyslipidemia	158	96.3	160	96.4	0.999
Microalbuminuria	102	62.2	100	60.2	

* Chi-square test.

**Table 3:** Secondary outcomes among two groups at baseline.

Types			Intervention Group		Control Group		*p* *
			n	%	n	%	
Risk behaviors/lifestyle	Low vegetable consumption (<400 g/day)	No	15	9.1	20	12.0	0.392
		Yes	149	90.9	146	88.0	
	High salt intake (>3 days/week)	No	56	34.1	56	33.7	0.937
		Yes	108	65.9	110	66.3	
	High consumption of fatty foods (>3 days/week)	No	101	61.6	101	60.8	0.890
		Yes	63	38.4	65	39.2	
	Alcohol consumption	Yes	24	14.6	25	15.1	0.913
		No	140	85.4	141	84.9	
	Smoking habit	Yes	38	23.2	32	19.3	0.387
		No	126	76.8	134	80.7	
	Low physical activity level	No	67	40.9	66	39.8	0.839
		Yes	97	59.1	100	60.2	
Outcomes	BMI controlled	Yes	92	56.1	85	51.2	0.373
		No	72	43.9	81	48.8	
	Treatment adherence (risk)	Yes	83	50.6	81	48.8	0.742
		No	81	49.4	85	51.2	
	Blood pressure controlled (outcome)	Yes	53	32.3	49	29.5	0.582
		No	111	67.7	117	70.5	

* Chi-square test. BMI: Body mass index.

**Table 4:** Outcome measures before and after intervention.

Outcomes	Intervention (n = 164) Control (n = 166)	Mean (SD)	
		Before	After
BMI	Intervention	22.9 (3.09)	22.38 (2.93)
	Control	22.89 (3.73)	23.31 (2.61)
Systolic BP	Intervention	128.60 (14.67)	128.60 (14.67)
	Control	141.16 (19.93)	141.16 (19.93)
Diastolic BP	Intervention	77.3 (10.45)	77.3 (10.45)
	Control	87.8 (11.51)	87.8 (11.51)
Total cholesterol	Intervention	5.28 (1.25)	5.28 (1.25)
	Control	5.68 (1.19)	5.68 (1.19)
Triglyceride	Intervention	2.63 (1.49)	2.63 (1.49)
	Control	2.82 (1.78)	2.82 (1.78)
Low-density lipoprotein (LDL)	Intervention	2.83 (1.09)	2.83 (1.09)
	Control	3.09 (1.04)	3.09 (1.04)
High-density lipoprotein (HDL)	Intervention	1.25 (0.33)	1.25 (0.33)
	Control	1.24 (0.25)	1.24 (0.25)
Microalbuminuria	Intervention	72.73 (87.87)	72.73 (87.87)
	Control	121.36 (74.18)	121.36 (74.18)

**Table 5:** Effectiveness in dietary risk habits, substance use, and physical activity level.

Outcomes		Before		After		*p* *	*p* **	ARR (%)	NNT (People)
		n	%	n	%				
Low vegetable consumption (<400 g/day)									
Intervention (n = 164)	No	15	9.1	47	28.7	0.144	<0.001	7	14
	Yes	149	90.9	117	71.3				
Control (n = 166)	No	20	12.0	36	21.7		0.007		
	Yes	146	88.0	130	78.3				
High salt intake (≥3 days/week)									
Intervention (n = 164)	No	56	34.1	92	56.1	0.002	<0.001	16.9	6
	Yes	108	65.9	72	43.9				
Control (n = 166)	No	56	33.7	65	39.2		0.342		
	Yes	110	66.3	101	60.8				
High fried, fatty food consumption (≥3 days/week)									
Intervention (n = 164)	No	101	61.6	125	76.2	0.077	0.001	8.3%	12
	Yes	63	38.4	39	23.8				
Control (n = 166)	No	101	60.8	112	67.5		0.200		
	Yes	65	39.2	54	32.5				
Alcohol consumption									
Intervention (n = 164)	Yes	24	14.6	17	10.4	0.085	0.167	6.5	15
	No	140	85.4	147	89.6				
Control (n = 166)	Yes	25	15.1	28	16.9		0.678		
	No	141	84.9	138	83.1				
Smoking habit									
Intervention (n = 164)	Yes	38	23.2	27	16.5	0.726	0.027	−0.06	-
	No	126	76.8	137	83.5				
Control (n = 166)	Yes	32	19.3	25	15.1		0.065		
	No	134	80.7	141	84.9				
Low physical activity									
Intervention (n = 164)	Yes	67	40.9	108	65.9	<0.001	<0.001	21.9	5
	No	97	59.1	56	34.1				
Control (n = 166)	Yes	66	39.8	73	44.0		0.419		
	No	100	60.2	93	56.0				

* Chi-square test; ** McNemar test. ARR: absolute risk reduction; NNT: number needed to treat (NNT).

**Table 6:** Effectiveness in BMI control, treatment adherence, and blood pressure control.

Outcomes		Before		After		*p* *	*p* **	ARR (%)	NNT (People)
		n	%	n	%				
BMI goal achieved									
Intervention	Yes	92	56.1	122	74.4	0.024	<0.001	13.6	7
	No	72	43.9	42	25.6				
Control	Yes	85	51.2	101	60.8		0.002		
	No	81	48.8	65	39.2				
Treatment adherence									
Intervention	Yes	83	50.6	153	93.3	<0.001	<0.001	31.3	3
	No	81	49.4	11	6.7				
Control	Yes	81	48.8	103	62.0		0.010		
	No	85	51.2	63	38.0				
Blood pressure goal achieved									
Intervention	Yes	53	32.3	111	67.7	<0.001	<0.001	27.9	3
	No	111	67.7	53	32.3				
Control	Yes	49	29.5	66	39.8		0.030		
	No	117	70.5	100	60.2				

* Chi-square test; ** McNemar test. ARR: absolute risk reduction; NNT: number needed to treat (NNT).

**Table 7:** Effectiveness of interventions on blood lipid profile and microalbumin.

Group	Goal Achieved				RR (CI 95%)	*p* *	ARR	NNT
	Yes		No					
	n	%	n	%				
Dyslipidemia								
Intervention (n = 158)	43	27.2	115	72.8	1.74 (1.12–2.70)	0.012	11.6	9
Control (n = 160)	25	15.6	135	84.4				
Microalbuminuria								
Intervention (n = 102)	45	44.1	57	55.9	1.52 (1.09–2.12)	0.007	17.9	6
Control (n = 103)	27	26.2	76	73.8				

* Chi-square test; RR: relative risk; CI: confidence interval; ARR: absolute risk reduction; NNT: number needed to treat (NNT).

**Table 8:** Incremental cost-effectiveness ratio.

Total Intervention Cost Difference between 2 Groups = 6627 USD in 2 Years			
Outcomes	Average Intervention Cost (USD)	Differences in Outcomes between 2 Groups (Patients)	Incremental Cost-Effectiveness Ratio (ICER)
Low vegetable consumption	607	16	38
High salt intake	607	27	22
High fried, fatty food consumption	607	13	47
Alcohol consumption	607	4	152
Smoking habit	607	4	152
Low physical activity	607	34	18
BMI goal achieved	607	14	43
Treatment adherence	607	48	13
Blood pressure goal achieved	607	41	15
Dyslipidemia goal achieved	607	18	34
Microalbuminuria goal achieved	607	18	34

## Data Availability

The data that support the findings of this study are available from the corresponding authors V.D.T. and T.T.P. (Trung Tin Pham) upon reasonable request.
